# Supplementary material for: Mechanistic understanding of human SLFN11
Source: Nat Commun. 2022 Sep 17;13:5464. doi: 10.1038/s41467-022-33123-0 (PMC9482658; doi:10.1038/s41467-022-33123-0)
Supplement: Supplementary file 1 — Supplementary Information [file 41467_2022_33123_MOESM1_ESM.pdf]

Supplementary Information

Mechanistic understanding of human SLFN11

Felix J. Metzner, Simon J. Wenzl, Michael Kugler, Stefan Krebs, Karl-Peter Hopfner and Katja Lammens

Supplementary Figures

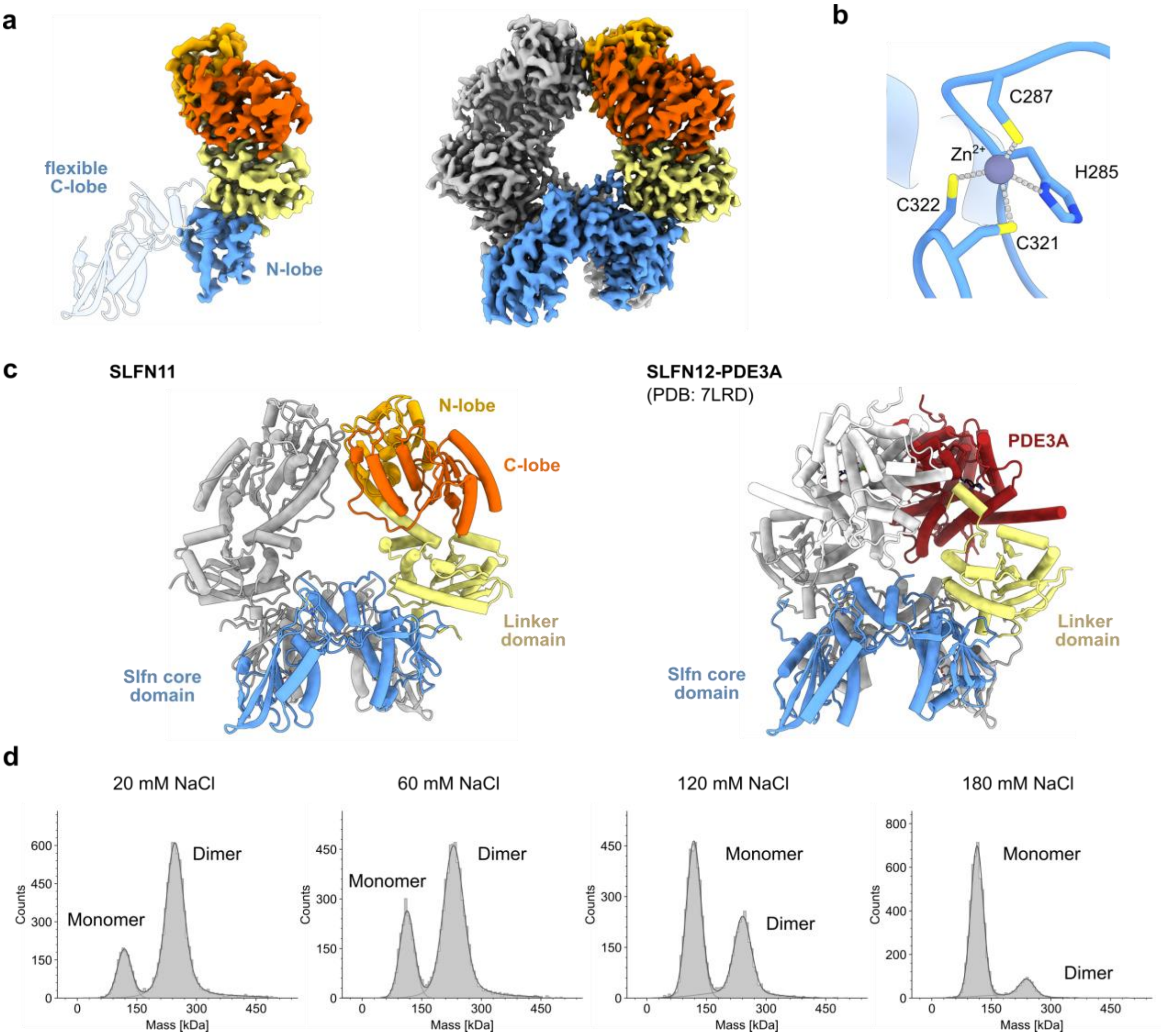

Supplementary Fig. 1: Analysis of SLFN11 monomeric and dimeric form. a, Cryo-EM reconstruction of the SLFN11^{E209A} monomer at 4.0 Å. The position of the missing C-lobe of Slfn core domain is depicted as a transparent cartoon model based on the SFLN11 dimeric structure. Cryo-EM reconstruction of the SLFN11^{E209A} dimer at 3.25 Å. **b**, Close-up view on SLFN11 zinc finger with depicted residues coordinating a zinc ion. **c**, Structural comparison of SLFN11 and SLFN12-PDE3A (PDB code 7LRD). The opposing second protomers of SLFN12 and PDE3A are coloured in grey and white, respectively. **d**, Mass distribution of SLFN11 monomers and dimers in the presence of variable concentrations of NaCl observed by mass photometry.

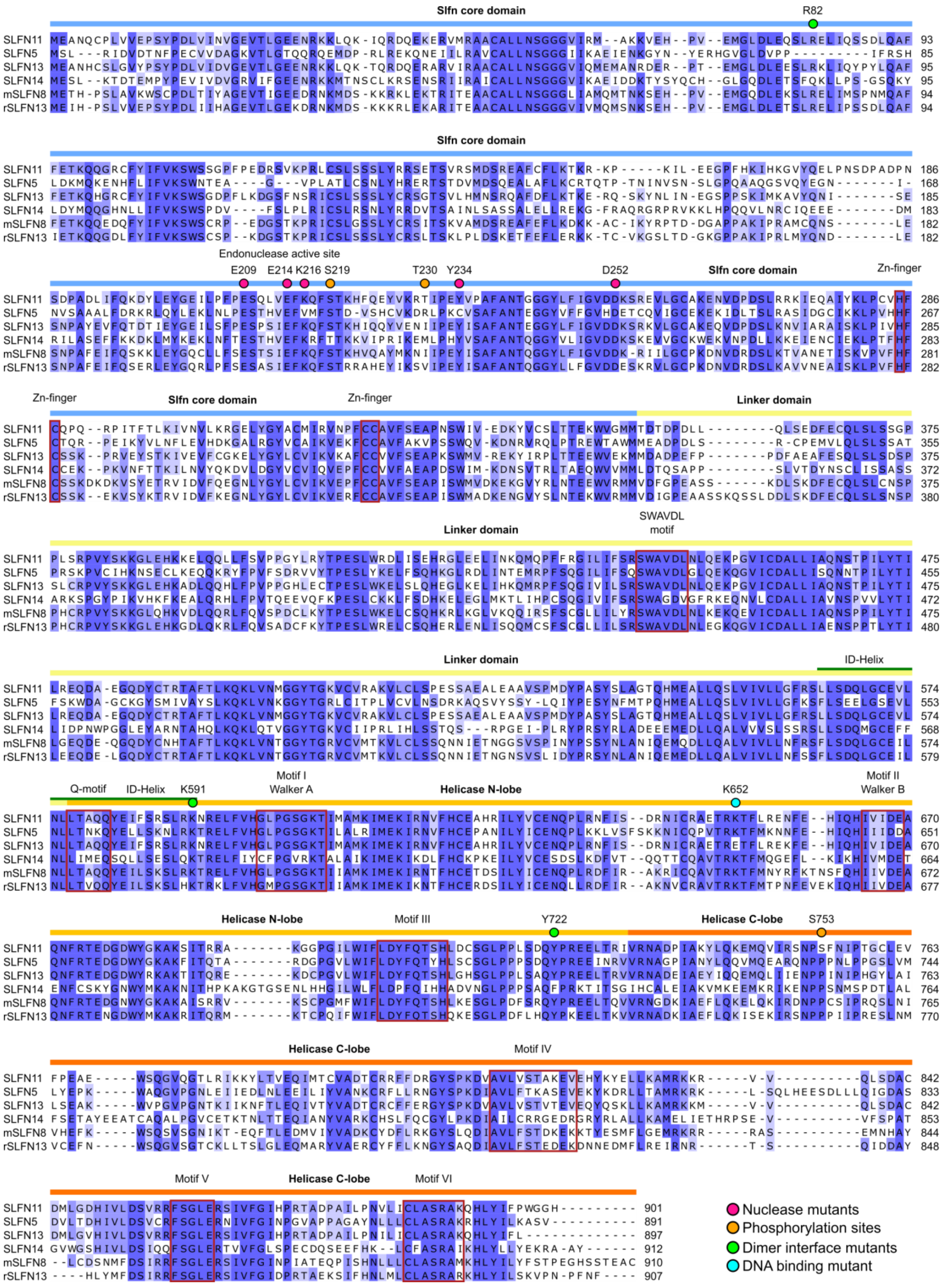

Supplementary Fig. 2: Multiple sequence alignment of SLFN11 and selected Schlafen proteins from human (SLFN5, SLFN13, SLFN14), mouse (mSLFN8) and rat (rSLFN13). The alignment was calculated using T-Coffee[1]. Residues are coloured according to percentage

identity (dark blue = more conserved, white = less conserved). The Slfn core domain is indicated in blue, the linker domain in yellow and the helicase domain in orange. The individual motifs are boxed in red squares. Mutated residues and phosphorylation sites are highlighted in circles in corresponding colour based on the legend.

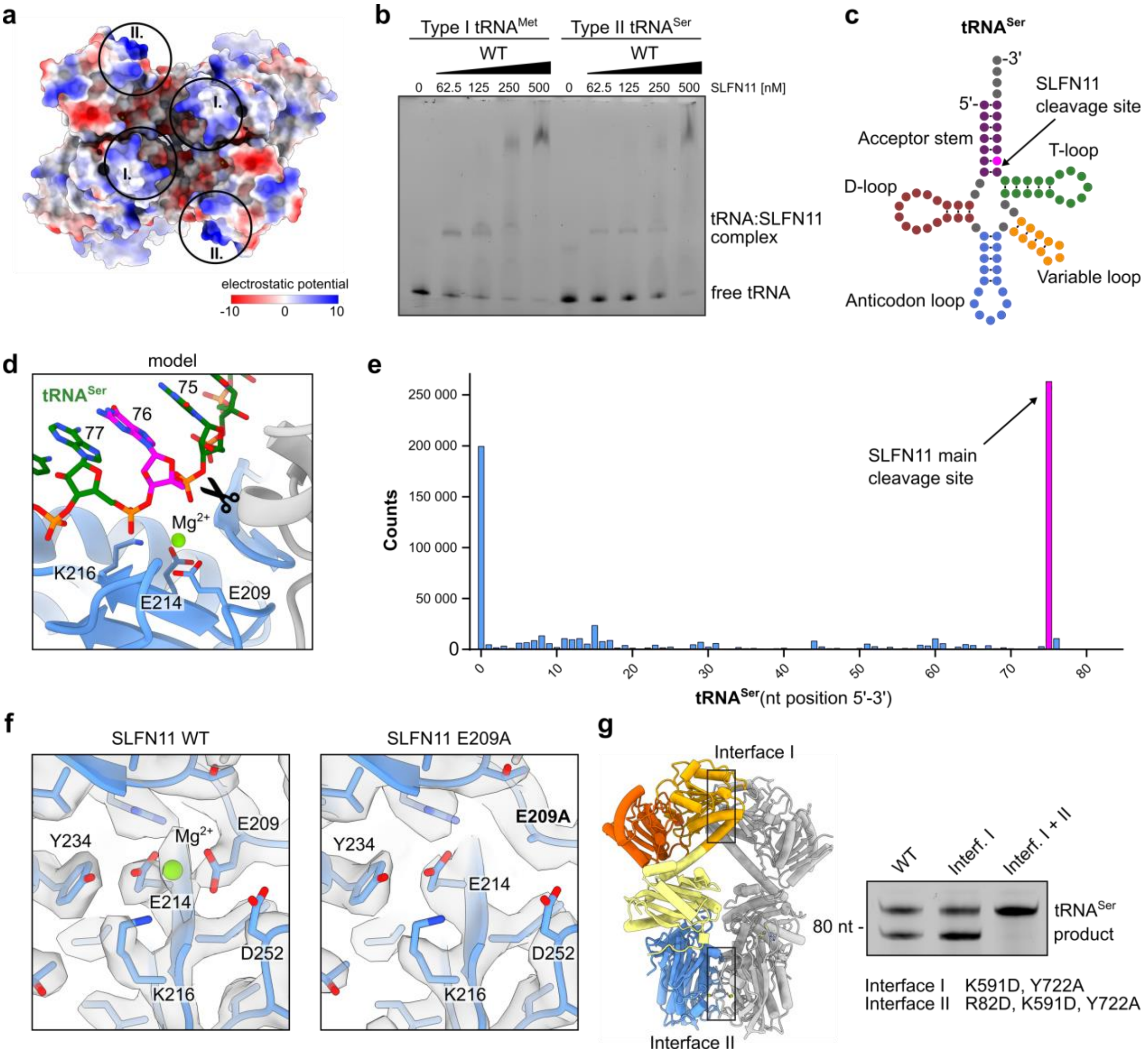

Supplementary Fig. 3: Binding of tRNA to SLFN11 and its regulation. a, Bottom view of the Slfn core domain shown as an electrostatic surface with positively charged patches (I. and II.) responsible for tRNA binding. The electrostatic potential values are in units of kcal mol^{-1} e^{-1} at 298 K. **b**, SLFN11 binding towards tRNA^{Met} and tRNA^{Ser} monitored by electrophoretic mobility shift assay. **c**, Schematic representation of tRNA^{Ser}. SLFN11 cleavage site is coloured in pink. **d**, Structural model of tRNA^{Ser} bound to SLFN11. Residues of the nuclease active site and the metal ion are shown. tRNA^{Ser} bases are numerically labelled from 5’ to 3’. The cleavage site between position 75 and 76 (pink) is indicated. **e**, Analysis of tRNA^{Ser} cleavage products by tRNA sequencing. Read counts are plotted against the start positions of the mapped sequencing reads. The main cleavage site between position 75 and 76 is indicated (pink). **f**, Comparison of SLFN11 WT and E209A mutant nuclease active sites with corresponding cryo-EM density map. **g**, Localization of interface I (K591D, Y722A) and interface II (R82D, K591D, Y722A) mutants of SLFN11 and their ability to cleave tRNA^{Ser} as observed in nuclease

assay. Experiments in **b**, **g** were performed in duplicates. One representative replicate is shown. Source data for **b**, **e**, **g** are provided as a Source Data file.

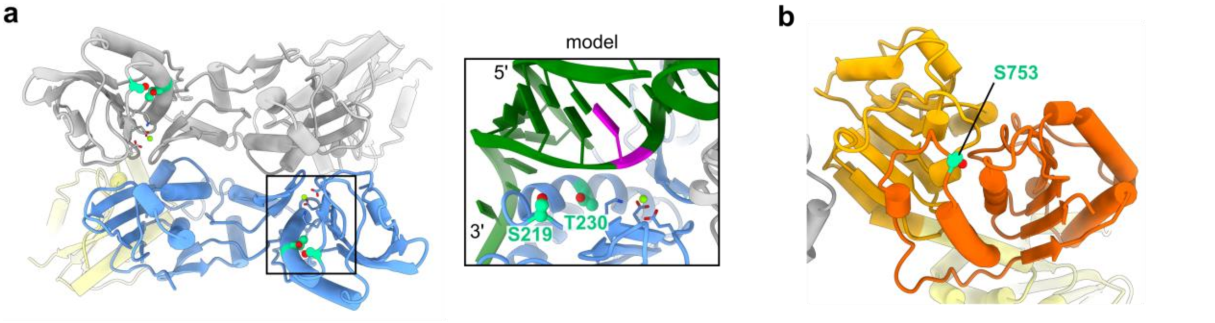

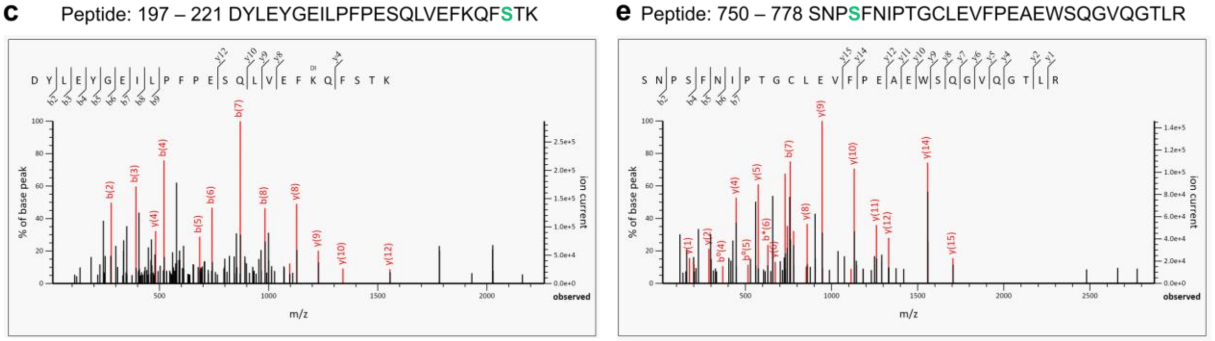

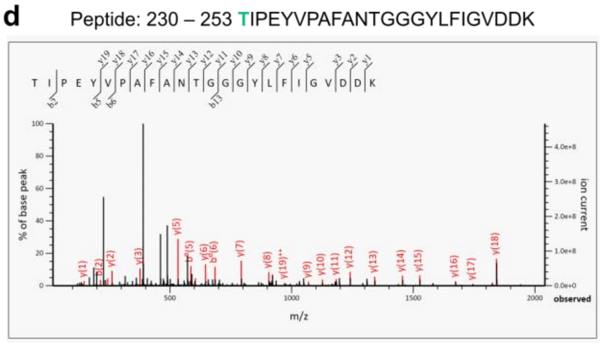

f

Peptide	Mr (exp.)	Mr (theor.)	ppm	Score	Expect
AA 197-221	3120.5321	3120.5332	-0.35	59	2.90E-06
AA 230-253	2543.2538	2543.2584	-1.82	78	4.90E-08
AA 750-778	3220.5221	3220.5135	2.66	40	0.00018

Supplementary Fig. 4: Phosphorylation sites of SLFN11. a, Left: Bottom view of Slfn core domains. SLFN11 phosphorylation sites S219 and T230 are coloured in light green. Nuclease active site residues are shown. Right: Structural model of $tRNA^{Ser}$ (green) bound to SLFN11. SLFN11 cleavage site is coloured in pink. Phosphorylation sites S219 and T230 are coloured in light green. Residues of the nuclease active site are shown. **b**, Position of phosphorylation site in SLFN11 helicase domain (S753). Residue S753 is shown and coloured in light green. **c**, MS/MS spectra of SLFN11 peptides AA 197–221 covering S219. **d**, MS/MS spectra of SLFN11 peptides AA 230-253 covering T230. **e**, MS/MS spectra of SLFN11 peptides AA 750-778 covering S753. **f**, Overview of Mascot[2] results including experimental (Mr exp.) and theoretical (Mr theor.) relative molecular mass, mass deviation in parts per million (ppm), Mascot ions scores (Score) and expectation values (Expect).

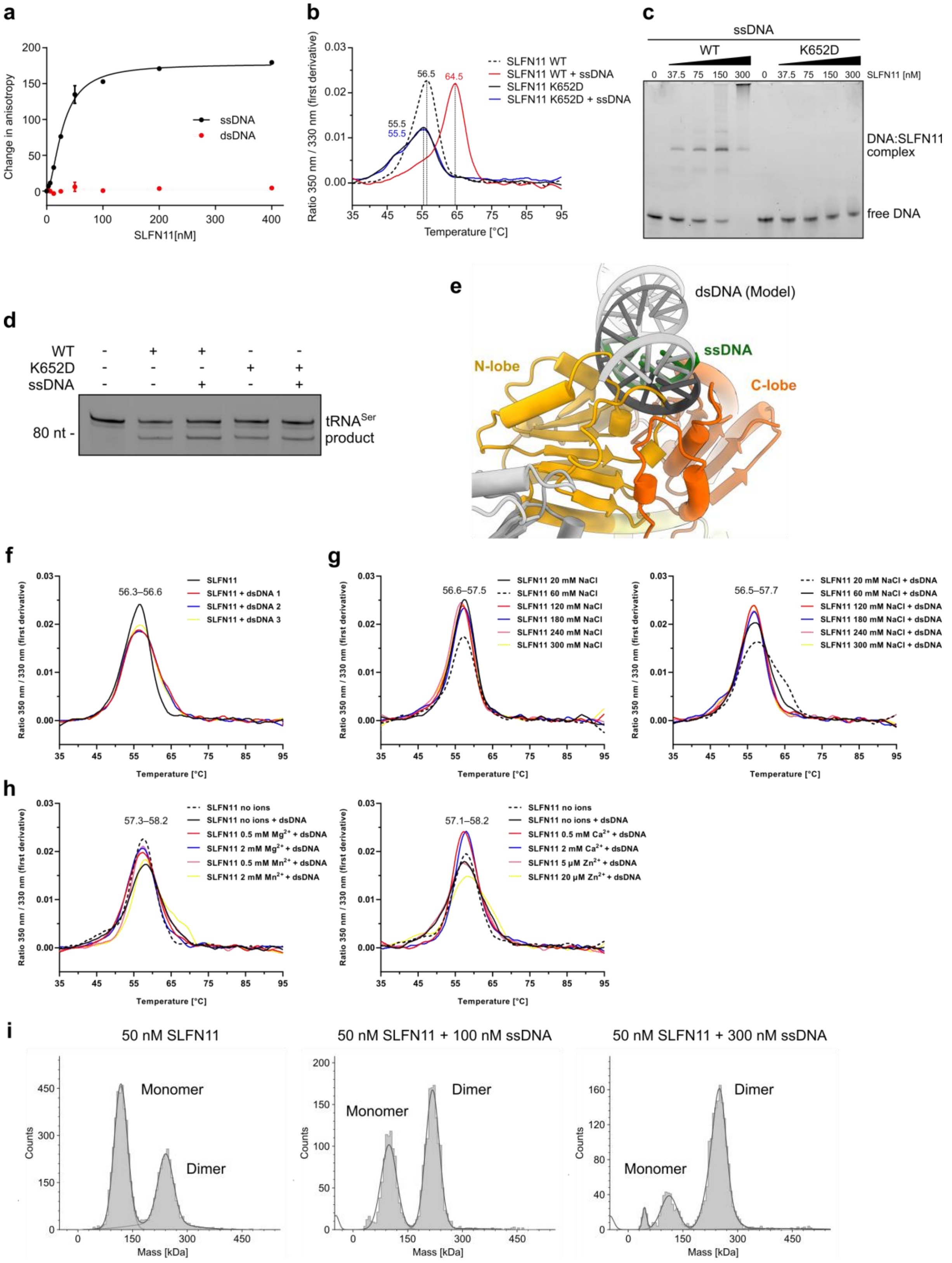

Supplementary Fig. 5: Characterization of DNA binding by SLFN11. a, Fluorescence anisotropy assay illustrating difference in binding ability of SLFN11 towards 50 nt ssDNA and 50 bp dsDNA. The data were fit to a cooperative binding equation. Data are represented as mean values +/- SEM from three independent experiments. **b**, NanoDSF measurements of

SLFN11^{wt} and SLFN11^{K652D} in the presence of 50 nt ssDNA. **c**, Abolished ssDNA binding ability of SLFN11 by single-point mutation K652D monitored by electrophoretic mobility shift assay. **d**, The effect of ssDNA-induced dimerization of SLFN11 on the nuclease activity of SLFN11 examined by nuclease assay. **e**, Structural model of dsDNA not fitting into the SLFN11 DNA-binding groove between the helicase N- and C-lobes. **f**, NanoDSF measurements of SLFN11^{wt} in the presence of 50 bp dsDNAs with different sequences. **g**, NanoDSF measurements of SLFN11^{wt} at different NaCl concentration without (left) or with (right) dsDNA present. **h**, NanoDSF measurements of SLFN11^{wt} in the presence of different bivalent ions (left: Mg^{2+}, Mn^{2+}; right; Ca^{2+}, Zn^{2+}) with or without dsDNA present. **i**, Stabilization of SLFN11 dimer by ssDNA observed in mass photometry. Experiments in **c**, **d** were performed in duplicates. One representative replicate is shown. Source data for **a**, **b**, **c**, **d**, **f**, **g**, **h** are provided as a Source Data file.

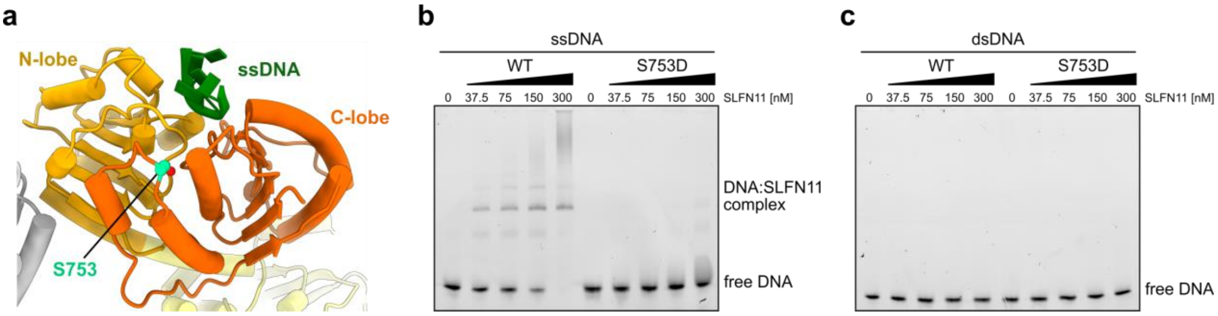

Supplementary Fig. 6: Characterization of SLFN11 phosphomimetic mutant S753D. a, Position of SLFN11 phosphorylation site in ssDNA bound helicase domain (S753). Residue S753 is shown and coloured in light green. **b**, Abolished ssDNA binding ability of SLFN11 by single-point mutation S753D monitored by electrophoretic mobility shift assay. **c**, dsDNA binding ability of SLFN11 by single-point mutation S753D monitored by electrophoretic mobility shift assay. Experiments in **b**, **c** were performed in duplicates. One representative replicate is shown. Source data for **b**, **c** are provided as a Source Data file.

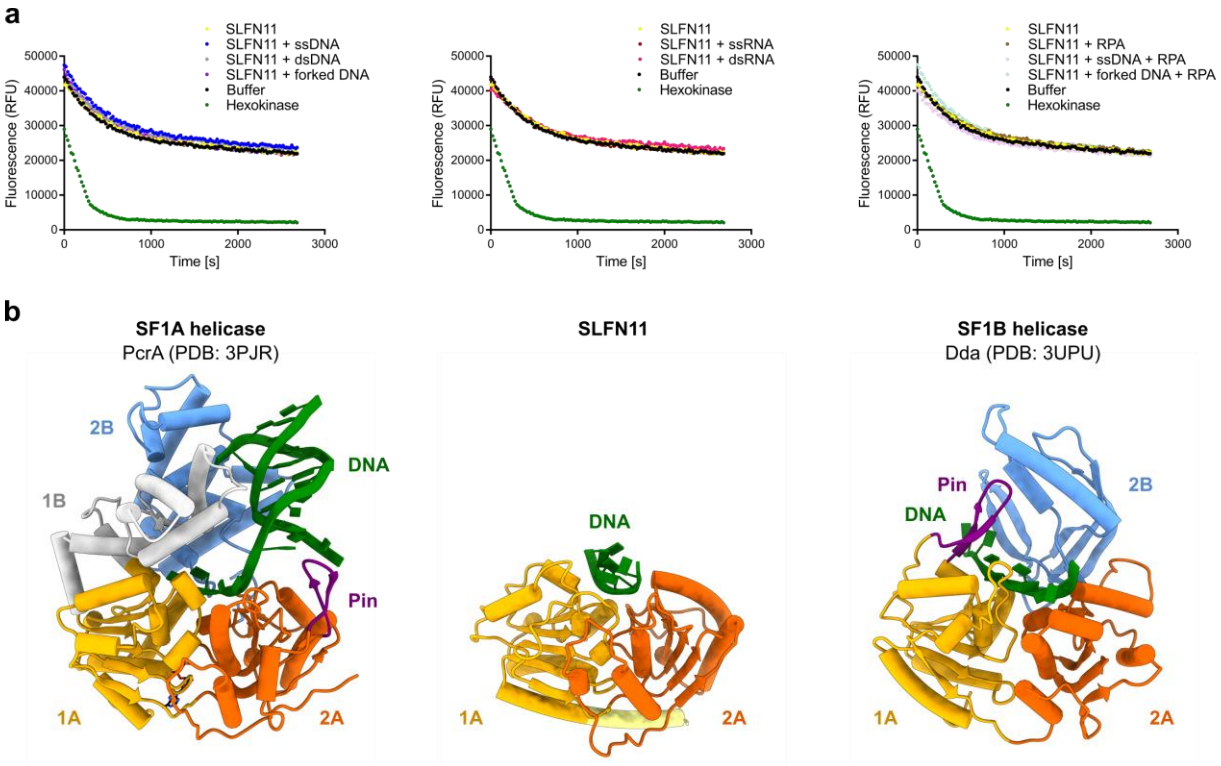

Supplementary Fig. 7: SLFN11 ATPase is locked in autoinhibited state. a, Fluorescence-based ATPase assay of SLFN11 (negative control: buffer; positive control: hexokinase). The fluorescence of NADH is measured over time. ATP consumption is enzymatically coupled to the oxidation of NADH, causing a decrease in fluorescence upon ATP hydrolysis. The details of individual substrates are shown in Supplementary Table 2. **b**, Structural comparison of SLFN11 helicase domain with PcrA SF1A helicase (PDB code 3PJR) and Dda helicase SF1B (PDB code 3UPU). The characteristic Pin in purple required for splitting of the incoming DNA duplex is missing in SLFN11. Source data for **a** are provided as a Source Data file.

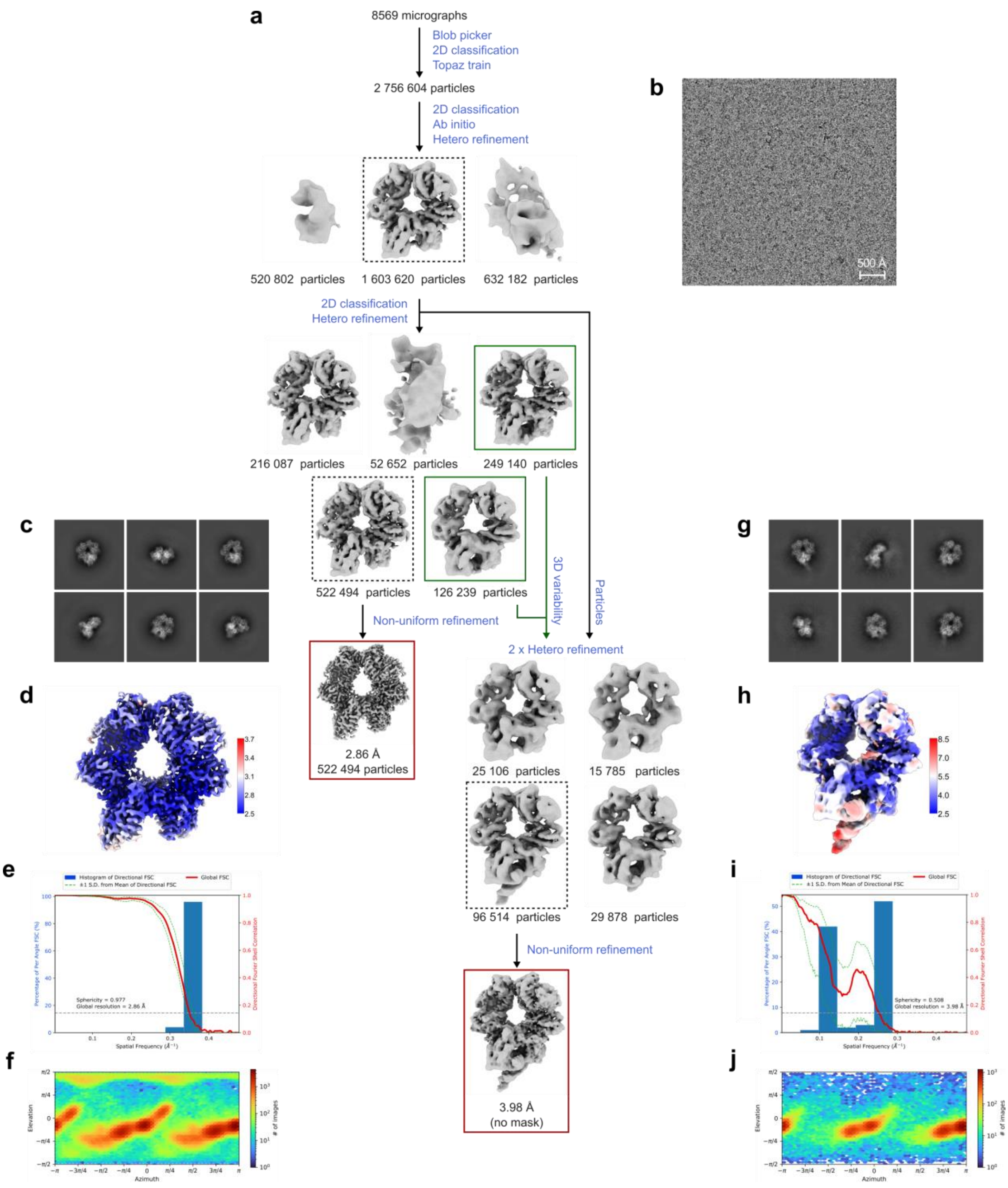

Supplementary Fig. 8: Cryo-EM data analysis of SLFN11^{wt} and SLFN11^{wt} with tRNA. **a**, Cryo-EM data processing workflow of SLFN11^{wt} and SLFN11^{wt} with tRNA using cryoSPARC[3]. **b**, Representative micrograph of SLFN11^{wt} and SLFN11^{wt} with tRNA dataset. The displayed micrograph is representative of 8,569 movies collected. **c**, Representative classes of a 2D classification of the particles used for the final SLFN11^{wt} reconstruction. **d**, Visualization of local resolution of SLFN11^{wt} calculated in cryoSPARC. Blue indicates higher resolution and red indicates lower resolution. **e**, Histogram of directional Gold-standard Fourier shell correlation (FSC)[4] (blue) and global FSC curve (red) of the final SLFN11^{wt} reconstruction.

The spread of directional resolution values is defined as ±1σ (dashed green lines). The grey dashed line indicates the 0.143 cutoff criterion, indicating a nominal resolution of 2.86 Å. **f**, Angular distribution of the particles used for the final SLFN11^{wt} reconstruction. **g**, Representative classes of a 2D classification of the particles used for the SLFN11^{wt} with tRNA reconstruction. **h**, Visualization of local resolution of SLFN11^{wt} with tRNA calculated in cryoSPARC. Blue indicates higher resolution and red indicates lower resolution. **i**, Histogram of directional FSC[4] (blue) and global FSC curve (red) of the final SLFN11^{wt} with tRNA reconstruction. The spread of directional resolution values is defined as ±1σ (dashed green lines). The grey dashed line indicates the 0.143 cutoff criterion, indicating a nominal resolution of 3.98 Å (no mask applied). **j**, Angular distribution of the particles used for the final SLFN11^{wt} with tRNA reconstruction.

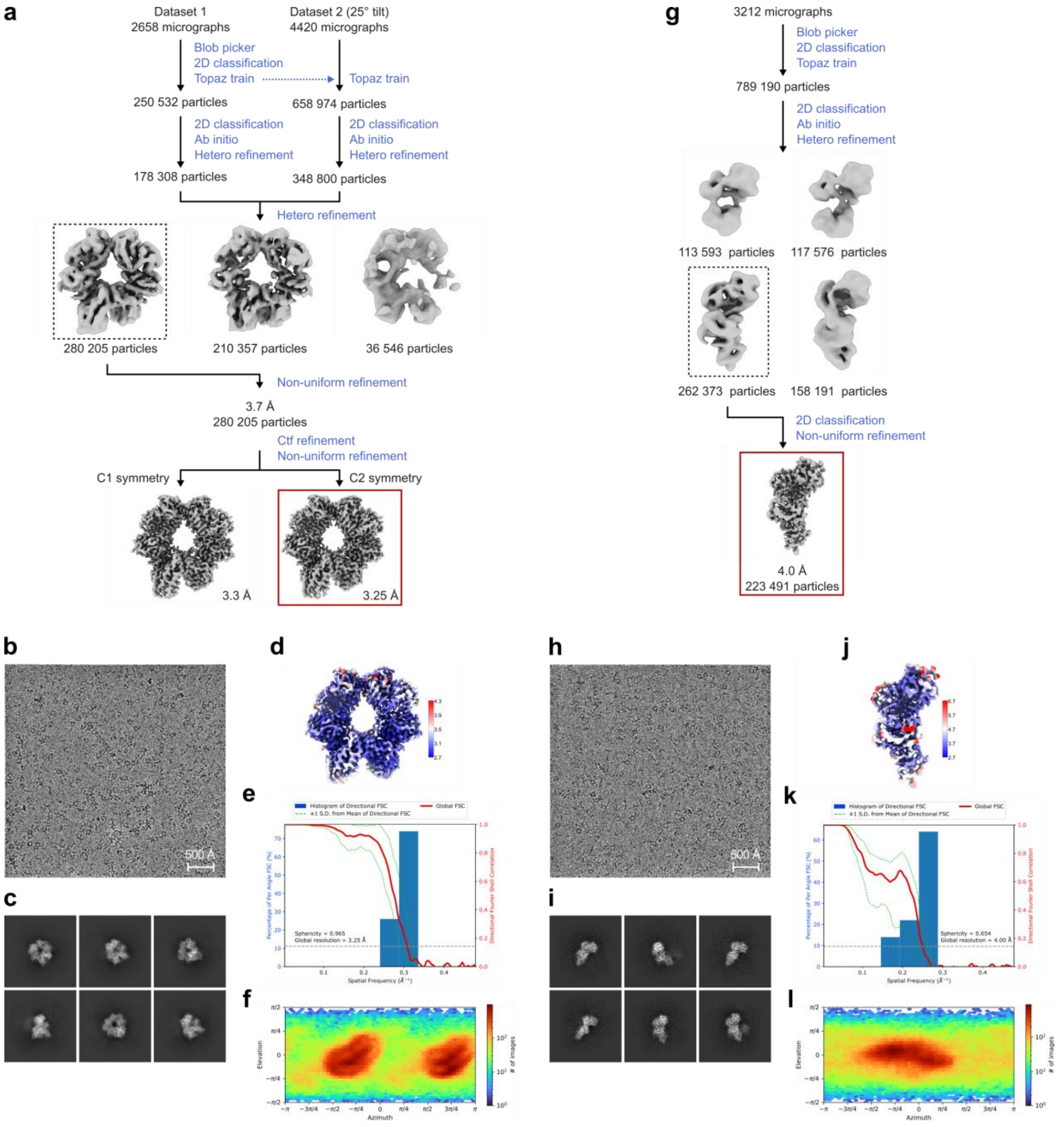

Supplementary Fig. 9: Cryo-EM data analysis of SLFN11^{E209A} dimer and monomer. **a**, Cryo-EM data processing workflow of SLFN11^{E209A} dimer using cryoSPARC[3]. **b**, Representative micrograph of SLFN11^{E209A} dimer. The displayed micrograph is representative of 7,078 movies collected. **c**, Representative classes of a 2D classification of the particles used for the final SLFN11^{E209A} dimer reconstruction. **d**, Visualization of local resolution of SLFN11^{E209A} calculated in cryoSPARC. Blue indicates higher resolution and red indicates lower resolution. **e**, Histogram of directional FSC[4] (blue) and global FSC curve (red) of the final SLFN11^{E209A} dimer reconstruction. The spread of directional resolution values is defined as ±1σ (dashed green lines). The grey dashed line indicates the 0.143 cutoff criterion, indicating a nominal resolution of 3.25 Å. **f**, Angular distribution of the particles used for the final

SLFN11^{E209A} dimer reconstruction. **g**, Cryo-EM data processing workflow of SLFN11^{E209A} monomer using cryoSPARC[3]. **h**, Representative micrograph of SLFN11^{E209A} monomer. The displayed micrograph is representative of 3,212 movies collected. **i**, Representative classes of a 2D classification of the particles used for the final SLFN11^{E209A} monomer reconstruction. **j**, Visualization of local resolution of SLFN11^{E209A} monomer calculated in cryoSPARC. Blue indicates higher resolution and red indicates lower resolution **k**, Histogram of directional FSC[4] (blue) and global FSC curve (red) of the final SLFN11^{E209A} monomer reconstruction. The spread of directional resolution values is defined as ±1σ (dashed green lines). The grey dashed line indicates the 0.143 cutoff criterion, indicating a nominal resolution of 4.0 Å. **l**, Angular distribution of the particles used for the final SLFN11^{E209A} monomer reconstruction.

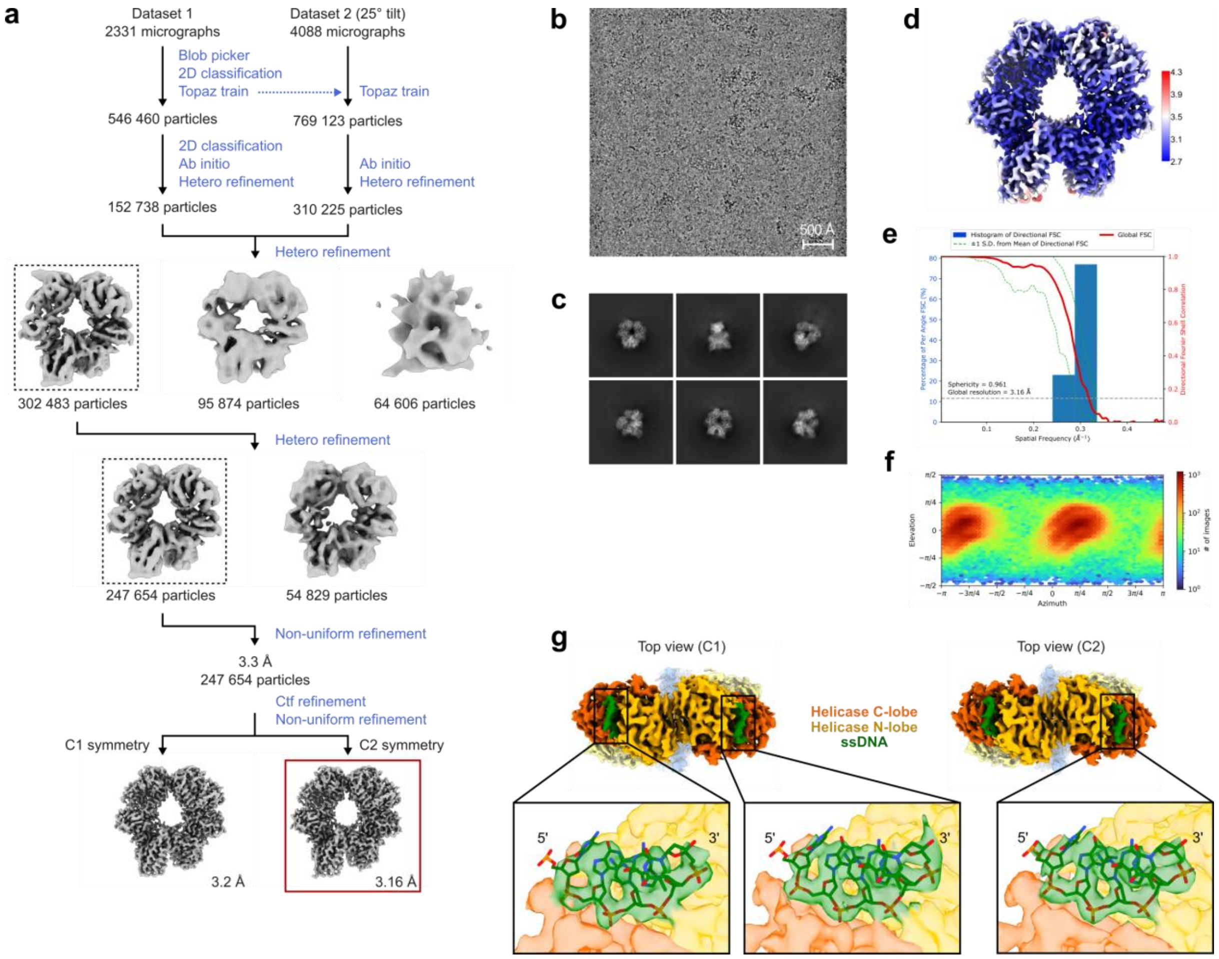

Supplementary Fig. 10: Cryo-EM data analysis of SLFN11^{wt} with ssDNA. a, Cryo-EM data processing workflow of SLFN11^{wt} with ssDNA using cryoSPARC[3]. **b**, Representative micrograph of SLFN11^{wt} with ssDNA. The displayed micrograph is representative of 6,419 movies collected. **c**, Representative classes of a 2D classification of the particles used for the final SLFN11^{wt} with ssDNA reconstruction. **d**, Visualization of local resolution of SLFN11^{wt} with ssDNA calculated in cryoSPARC. Blue indicates higher resolution and red indicates lower resolution. **e**, Histogram of directional FSC[4] (blue) and global FSC curve (red) of the final SLFN11^{wt} with ssDNA reconstruction. The spread of directional resolution values is defined as ±1σ (dashed green lines). The grey dashed line indicates the 0.143 cutoff criterion, indicating a nominal resolution of 3.16 Å. **f**, Angular distribution of the particles used for the final SLFN11^{wt} with ssDNA reconstruction. **g**, Top views of cryo-EM reconstructions of SLFN11^{wt} with ssDNA with C1 (left) or C2 (right) symmetry imposed. Helicase lobes and DNA are colour coded. Bottom: Detailed views of the cryo-EM densities around the ssDNA for the C1 (left) and C2 (right) reconstructions.

Supplementary Tables

Supplementary Table 1: Cryo-EM data collection, refinement and validation statistics.

	SLFN11 WT (EMD-14690) (PDB 7ZEL)	SLFN11 E209A dimer (EMD-14691) (PDB 7ZEP)	SLFN11 E209A monomer (EMD-14693)	SLFN11 WT + ssDNA (EMD-14692) (PDB 7ZES)	SLFN11 WT + tRNA (EMD-14695)
**Data collection and processing**					
Magnification	130,000	130,000	130,000	130,000	130,000
Voltage (kV)	300	300	300	300	300
Electron exposure (e^{-}/Å^2)	49.7	44.0	43.6	43.3	49.7
Defocus range (μm)	-1.1 to -2.9	-1.1 to -2.9	-1.1 to -2.9	-1.1 to -2.9	-1.1 to -2.9
Pixel size (Å)	1.046	1.046	1.046	1.046	1.046
Symmetry imposed	C1	C2	C1	C2	C1
Initial particle images (no.)	2,756,604	909,506	789,190	1,315,583	2,756,604
Final particle images (no.)	522,494	280,205	223,491	247,654	96,514
Map resolution (Å)	2.86	3.25	4.0	3.16	3.98
FSC threshold	0.143	0.143	0.143	0.143	0.143
**Refinement**					
Initial model used	AlphaFold	AlphaFold		AlphaFold	
Map sharpening *B* factor (Å^2)	132.7	157.3		146.8	
Model composition					
Non-hydrogen atoms	13,334	13,324		13,538	
Protein residues	1,654	1,654		1,654	
Ligands	2 Mg, 2 Zn	2 Zn		2 Mg, 2 Zn	
Nucleotide				10	
B factors (Å^2)					
Protein	42.40	52.29		42.02	
Ligand	57.56	91.95		57.92	
Nucleotide				84.10	
R.m.s. deviations					
Bond lengths (Å)	0.003	0.002		0.005	
Bond angles (°)	0.569	0.475		0.609	
Validation					
MolProbity score	1.25	1.27		1.39	
Clashscore	4.85	5.15		5.90	
Poor rotamers (%)	0.00	0.00		0.00	
Ramachandran plot					
Favored (%)	98.41	98.29		97.68	
Allowed (%)	1.59	1.71		2.32	
Disallowed (%)	0.00	0.00		0.00	

Supplementary Table 2: List of oligonucleotides (5’ to 3’).

Purpose	Name	Sequence	Modification
Cloning	SLFN11_E209A_fwd	CCATTTCCCGCCAGCCAGCTCGTG	
	SLFN11_E209A_rev	CACGAGCTGGCTGGCGGGAAATGG	
	SLFN11_E214A_fwd	CCAGCTCGTGGCCTTCAAGCAGTTTTCC	
	SLFN11_E214A_rev	GGAAAACTGCTTGAAGGCCACGAGCTGG	
	SLFN11_K216A_fwd	CTCGTGGAGTTCGCGCAGTTTTCCACC	
	SLFN11_K216A_rev	GGTGGAAAACTGCGCGAACTCCACGAG	
	SLFN11_Y234A_fwd	CAATCCCTGAGGCGGTGCCAGCCTTC	
	SLFN11_Y234A_rev	GAAGGCTGGCACCGCCTCAGGGATTG	
	SLFN11_D252A_fwd	GTTTATCGGCGTGGACGCGAAGAGCAGAGAGGTG	
	SLFN11_D252A_rev	CACCTCTCTGCTCTTCGCGTCCACGCCGATAAAC	
	SLFN11_K652D_fwd	GCCGAGACAAGAGACACATTCCTGCGG	
	SLFN11_K652D_rev	CCGCAGGAATGTGTCTCTTGTCTCGGC	
	SLFN11_R82D_fwd	GAGCAGAGCCTGGATGAGCTGATCCAG	
	SLFN11_R82D_rev	CTGGATCAGCTCATCCAGGCTCTGCTC	
	SLFN11_K591D_fwd	CGGTCCCTGAGAGATAACAGGGAGCTG	
	SLFN11_K591D_rev	CAGCTCCCTGTTATCTCTCAGGGACCG	
	SLFN11_Y722A_fwd	CTGTCTGACCAGGCTCCCAGGGAGGAG	
	SLFN11_Y722A_rev	CTCCTCCCTGGGAGCCTGGTCAGACAG	
	SLFN11_S753D_fwd	CGCTCTAACCCTGACTTCAATATCCC	
	SLFN11_S753D_rev	GGGATATTGAAGTCAGGGTTAGAGCG	
Biochemistry	$tRNA^{Met}$	AGUAAGGUCAGCUAAAUAAGCUAUCGGGCCCAUACCCCGAAAAUGU UGGUUAUACCCUUCCCGUACUACCA	5´-FAM
	$tRNA^{Ser}$	GUAGUCGUGGCCGAGUGGUUAAGGCGAUGGACUUGAAAUCCAUUGU GGUUUCCCCGCGCAGGUUCGAAUCCUGCCGACUACGCCA	5´-FAM
	ssDNA 50 nt	TCTTTTTTTTTTTTGTTCTTTTTTTTGATTCCGGTTTCTTTGAAATTTTT	5´-FAM
	dsDNA 50 bp	TCTTTTTTTTTTTTGTTCTTTTTTTTGATTCCGGTTTCTTTGAAATTTTT	5´-FAM
	dsDNA 2 50 bp	TGTCTACTTGAAATTCTAATTCATATTTTTTTTGTTTGGATAGAATATCA	
	dsDNA 3 50 bp	CAAAAGTGACATCCACAGCAAGCTGGACAGGTAAATTGCCTCATACAA TC	
	ssDNA 60 nt EM	CGCGTACGTGCGTTTAGAGCTTGCTACGTGCGTTTAAGCGGTGCTAGA GCTTGCTACGAC	
	ssDNA 60 nt MF	CGCGTACGTGCGTTTAGAGCTTGCTACGTGCGTTTAAGCGGTGCTAGA GCTTGCTACGAC	
	ssDNA 60 nt ATPase	CGCGTACGTGCGTTTAGAGCTTGCTACGTGCGTTTAAGCGGTGCTAGA GCTTGCTACGAC	
	dsDNA 60 bp ATPase	CGCGTACGTGCGTTTAGAGCTTGCTACGTGCGTTTAAGCGGTGCTAGA GCTTGCTACGAC	
	ssDNA 50 nt (Fig. 3e)	AATTGGTCGTAGCAAGCTCTAGCACCGCTTAAACGCACGTACGCGCTG TC	5´-FAM
	forked DNA	$(dT)_{40}$CTACGACCAATTGAGCGGCCTCGGCACCGGGATTCTCCAG	5´-Cy5
	ssRNA 41 nt	GACGGCCAUACCACCCUGAACGGCCCGAUCUUGUCUGAUCC	5´-FAM
	dsRNA 41 bp	GACGGCCAUACCACCCUGAACGGCCCGAUCUUGUCUGAUCC	5´-FAM

References

1 Notredame, C., Higgins, D. G. & Heringa, J. T-Coffee: A novel method for fast and accurate multiple sequence alignment. *J. Mol. Biol.* **302**, 205-217 (2000).

2 Perkins, D. N., Pappin, D. J., Creasy, D. M. & Cottrell, J. S. Probability-based protein identification by searching sequence databases using mass spectrometry data. *Electrophoresis* **20**, 3551-3567 (1999).

3 Punjani, A., Rubinstein, J. L., Fleet, D. J. & Brubaker, M. A. cryoSPARC: algorithms for rapid unsupervised cryo-EM structure determination. *Nat. Methods* **14**, 290-296 (2017).

4 Tan, Y. Z. *et al.* Addressing preferred specimen orientation in single-particle cryo-EM through tilting. *Nat. Methods* **14**, 793-796 (2017).
