# Supplementary material for: Mechanistic understanding of human SLFN11
Source: Nat Commun. 2022 Sep 17;13:5464. doi: 10.1038/s41467-022-33123-0 (PMC9482658; doi:10.1038/s41467-022-33123-0)
Supplement: Supplementary file 3 — Reporting Summary [file 41467_2022_33123_MOESM3_ESM.pdf]

Corresponding author(s): Katja Lammens
Last updated by author(s): Aug 24, 2022

Reporting Summary

Nature Portfolio wishes to improve the reproducibility of the work that we publish. This form provides structure for consistency and transparency in reporting. For further information on Nature Portfolio policies, see our Editorial Policies and the Editorial Policy Checklist.

Statistics

For all statistical analyses, confirm that the following items are present in the figure legend, table legend, main text, or Methods section.

n/a	Confirmed	
☐	☒	The exact sample size (n) for each experimental group/condition, given as a discrete number and unit of measurement
☐	☒	A statement on whether measurements were taken from distinct samples or whether the same sample was measured repeatedly
☒	☐	The statistical test(s) used AND whether they are one- or two-sided<br>*Only common tests should be described solely by name; describe more complex techniques in the Methods section.*
☒	☐	A description of all covariates tested
☒	☐	A description of any assumptions or corrections, such as tests of normality and adjustment for multiple comparisons
☐	☒	A full description of the statistical parameters including central tendency (e.g. means) or other basic estimates (e.g. regression coefficient) AND variation (e.g. standard deviation) or associated estimates of uncertainty (e.g. confidence intervals)
☒	☐	For null hypothesis testing, the test statistic (e.g. F, t, r) with confidence intervals, effect sizes, degrees of freedom and P value noted<br>*Give P values as exact values whenever suitable.*
☒	☐	For Bayesian analysis, information on the choice of priors and Markov chain Monte Carlo settings
☒	☐	For hierarchical and complex designs, identification of the appropriate level for tests and full reporting of outcomes
☒	☐	Estimates of effect sizes (e.g. Cohen's d, Pearson's r), indicating how they were calculated

Our web collection on statistics for biologists contains articles on many of the points above.

Software and code

Policy information about availability of computer code

Data collection	EPU 2.12.1.278REL, TEM User interface Titan 2.15.4, Digital Micrograph 3.22.1461.0
Data analysis	MotionCor2 1.4.5, cryoSPARC 3.3.1, Phenix 1.17, ISOLDE 1.2.2, AlphaFold2, Coot 0.9-pre, UCSF ChimeraX 1.2, Prism 6.07, GIMP 2.10.2, ImageJ 1.8.0, Tycho NT.6 software 1.3.2.878, AcquireMP (Refeyn) 2.3, Burrows-Wheeler Aligner 0.17.7, MASCOT 2.4

For manuscripts utilizing custom algorithms or software that are central to the research but not yet described in published literature, software must be made available to editors and reviewers. We strongly encourage code deposition in a community repository (e.g. GitHub). See the Nature Portfolio guidelines for submitting code & software for further information.

Data

Policy information about availability of data

All manuscripts must include a data availability statement. This statement should provide the following information, where applicable:

- Accession codes, unique identifiers, or web links for publicly available datasets
- A description of any restrictions on data availability
- For clinical datasets or third party data, please ensure that the statement adheres to our policy

The coordinates of the SLFN11wt, SLFN11 E209A dimer, and SLFN11wt ssDNA-bound structure have been deposited in the Protein Data Bank (PDB) under the accession code 7ZEL [http://doi.org/10.2210/pdb7zel/pdb], 7ZEP [http://doi.org/10.2210/pdb7zep/pdb], and 7ZES [http://doi.org/10.2210/pdb7zes/pdb], respectively. The SLFN11wt dimer cryo-EM reconstruction is available at the Electron Microscopy Data Bank (EMDB) under the EMBD accession code EMD-14690 [https://www.ebi.ac.uk/emdb/entry/EMD-14690]. The SLFN11 dimer reconstruction of SLFN11wt bound to tRNA is available at the EMDB under the EMDB accession code EMD-14695 [https://www.ebi.ac.uk/emdb/entry/EMD-14695]. The SLFN11 monomer and dimer reconstruction of SLFN11 E209A is available at the EMDB under the EMDB accession code EMD-14693 [https://www.ebi.ac.uk/emdb/entry/EMD-14693] and EMD-14691 [https://www.ebi.ac.uk/emdb/entry/

EMD-14691], respectively. The SLFN11 dimer reconstruction of SLFN11wt bound to ssDNA is available at the EMDB under the accession code EMD-14692 [https://www.ebi.ac.uk/emdb/entry/EMD-14692]. MS spectra were searched using the human subset of the Swiss-Prot database [https://www.uniprot.org/]. Source data are provided with this paper.

Field-specific reporting

Please select the one below that is the best fit for your research. If you are not sure, read the appropriate sections before making your selection.

☒ Life sciences ☐ Behavioural & social sciences ☐ Ecological, evolutionary & environmental sciences

For a reference copy of the document with all sections, see nature.com/documents/nr-reporting-summary-flat.pdf

Life sciences study design

All studies must disclose on these points even when the disclosure is negative.

Sample size	No statistical methods were used to predetermine sample size. Sample sizes were chosen based on previous experience and the anticipated variance to obtain statistical significance and reproducibility. Size of cryo-EM dataset sample size was based on sufficient number of images and particles to obtain a high resolution reconstruction.
Data exclusions	No data were excluded.
Replication	Experiments that led to quantitative conclusions were performed in independent replicates as described in the figure legends.
Randomization	No statistical calculation was involved that require randomization.For cryo-EM analyses, particles were randomly assigned to half-maps for resolution determination following the standard procedures in cryoSPARC.
Blinding	No blinding was perfomed in order to avoid errors in sample naming.

Reporting for specific materials, systems and methods

We require information from authors about some types of materials, experimental systems and methods used in many studies. Here, indicate whether each material, system or method listed is relevant to your study. If you are not sure if a list item applies to your research, read the appropriate section before selecting a response.

Materials & experimental systems

n/a	Involved in the study	
☒	☐	Antibodies
☐	☒	Eukaryotic cell lines
☒	☐	Palaeontology and archaeology
☒	☐	Animals and other organisms
☒	☐	Human research participants
☒	☐	Clinical data
☒	☐	Dual use research of concern

Methods

n/a	Involved in the study	
☒	☐	ChIP-seq
☒	☐	Flow cytometry
☒	☐	MRI-based neuroimaging

Eukaryotic cell lines

Policy information about cell lines

Cell line source(s)	Spodoptera frugiperda Sf21 insect cells (Thermo Fisher, 11497013)<br>Trichoplusia ni High Five insect cells (Invitrogen, B85502)
Authentication	No methods were used for authentication.
Mycoplasma contamination	Protein expression cell lines were not tested.
Commonly misidentified lines<br>(See ICLAC register)	No commonly misidentified cell lines were used.
